# Sexual Satisfaction in Fully Ambulatory People with Multiple Sclerosis: Does Disability Matter?

**DOI:** 10.1155/2020/8857516

**Published:** 2020-10-10

**Authors:** Pawel Dobrakowski, Agnieszka Machowska-Majchrzak, Beata Labuz-Roszak, Ewa Niewiadomska, Krystyna Pierzchala

**Affiliations:** ^1^Faculty of Medical Sciences in Zabrze, Department of Neurology, Medical University of Silesia in Katowice, Poland; ^2^Faculty of Health Sciences in Bytom, Department of Basic Medical Sciences, Medical University of Silesia in Katowice, Poland; ^3^Faculty of Health Sciences in Bytom, Department of Epidemiology and Biostatistics, Medical University of Silesia in Katowice, Poland

## Abstract

Primary sexual dysfunctions (SD) are a direct result of neurological changes that affect the sexual response. Secondary SD result from the symptoms that do not directly involve nervous pathways to the genital system, such as bladder and bowel problems, fatigue, spasticity, or muscle weakness. Tertiary SD are the result of disability-related psychosocial and cultural issues that can interfere with sexual feelings and experiences. The aim of this study was to assess the sexual satisfaction (SS) in people with multiple sclerosis (PwMS) without significant mobility impairment and to estimate the influence of SD, the score on the Kurtzke Expanded Disability Status Scale (EDSS), lowered mood, and stress coping strategies on SS. *Methods*. 76 PwMS with the EDSS score < 5.0 points were enrolled in the study. The subjects completed the Montgomery-Asberg Depression Scale (MADRS), the Coping Inventory for Stressful Situations (CISS), the Multiple Sclerosis Intimacy and Sexuality Questionnaire (MSISQ-19), and the Sexual Satisfaction Questionnaire (SSQ). *Results*. The level of SS in PwMS was not significantly lower compared to that of the general population. It correlated with the primary, secondary, and tertiary SD and lowered mood. However, it did not correlate with disability measured by the EDSS. *Conclusions*. The level of SS in PwMS with the EDSS score below 5.0 points was not significantly lower. SS depended on SD, lowered mood, and stress coping style, and it was not significantly related to the level of disability in patients with the EDDS score below 5.0.

## 1. Introduction

Multiple sclerosis (MS) is a chronic inflammatory demyelinating disease of the central nervous system (CNS) that results in demyelination with axonal damage (white matter damage) and cerebral cortical atrophy (grey matter damage) [1]. It leads to progressive disability. The first signs of the disease usually occur in young adults between 20 and 40 years of age. Most of the patients are women [2]. The number of people with multiple sclerosis (PwMS) worldwide exceeds 2.5 million with approximately 630,000 cases reported in Europe. Poland is among the countries with a high incidence of MS. It is assumed that the incidence of MS in Poland ranges from 45 to 92 people per 100,000 inhabitants [3].

In most patients, clinical manifestations indicate the involvement of motor, sensory, visual, and autonomic systems. However, many other symptoms and signs can also occur. Only a few of the clinical features are disease specific. Lhermitte's symptom (an electric sensation running down the spine or limbs on neck flexion) and the Uhthoff phenomenon (transient worsening of symptoms and signs when the core body temperature increases, such as after exercise or a hot bath) are particularly characteristic [4].

Some impairment can result in feelings of depression, anxiety, low self-esteem/self-image, mood disorders, and partnership-related difficulties, which can lead to sexual dysfunction (SD) [5, 6]. Recent studies have indicated that the problem affects 40-80% of women and 50-90% of men [7–9]. MS male patients may develop erectile dysfunction, bladder dysfunction, and low libido. Women usually report diminished libido, reduced ability to experience pleasure, bladder dysfunctions, and fatigue due to MS [8, 10, 11]. Lower SS is related to the duration of the disease. It is possible that the longer the disease duration, the more psychological and relational problems are experienced by patients, including lower self-esteem, considering the fact that approximately 60% of patients changed the manner they perceived themselves after the diagnosis of MS [12].

The etiology of SD in MS is still a matter of debate [13]. However, it has been assumed that growing physical impairment, psychological factors, and drug-related adverse effects increase the rates of SD [14]. SD can develop at various stages of MS, starting at an early stage of the disease [15, 16] with a growing prevalence [17].

Foley and Iverson distinguished primary SD (directly related to demyelinating lesions), secondary SD (involving physical limitations, not directly related to the genital system), and tertiary SD (involving the influence of psychological, social, economic, and cultural aspects) [18]. Primary SD is directly related to demyelinating lesions in the spinal cord. The spinal cord is frequently affected in MS, causing motor, sensory, and autonomic dysfunction. A number of pathological abnormalities, including demyelination and neuroaxonal loss, are found on magnetic resonance imaging (MRI) [19].

SS is perceived as a significant part of the general quality of life and the quality of the relationship with a partner [20, 21]. Scholars agree with the fact that SS cannot be defined only in terms of orgasm. Pinney et al. consider SS a subjective evaluation of the level of sex life satisfaction [22]. Studies conducted by McCabe demonstrated that there was no simple correlation between the occurrence of SD and SS achieved by PwMS [23]. The author indicated that different strategies for coping with difficult situations were the cause of these discrepancies [24].

The aim of the study was to check whether SS in PwMS was directly related to disability measured on the EDSS and the presence of SD.

## 2. Material and Methods

### 2.1. Study Design

Seventy-six patients (46 women, 30 men) aged 21-54 years, diagnosed with MS according to the 2010 McDonald criteria, participated in the study [25, 26]. The subjects were the patients of the MS department in Zabrze, of whom 85.5% were diagnosed with relapsing-remitting MS (RRMS). Over 80% of patients were treated with immunomodulatory drugs. The mean time from diagnosis to study participation was 6.8 ± 4.6 years. Most patients were married. Six subjects (3 men and 3 women) declared that they were not involved in any relationship and therefore did not complete the Sexual Satisfaction Questionnaire (SSQ) (Table 1).

The information obtained from the medical history of patients included demographic data, treatment, the course of the disease, and mental status. The assessment of the neurological status and the level of disability (EDSS) was performed by a certified neurologist specializing in the EDSS [27]. The scale assesses individual functions of the CNS (pyramidal, cerebellar, brainstem, sensory, bowel, bladder, visual, cerebral, and mental functions). The scale includes the presence and intensity of symptoms. The scores range from 0 (normal) to 10 (death due to MS).

The participants were also assessed using the EDSS and the Montgomery-Asberg Depression Scale (MADRS) [28]. They also completed the Coping Inventory for Stressful Situations (CISS) [29], the Multiple Sclerosis Intimacy and Sexuality Questionnaire (MSISQ-19) [30], and the SSQ [31].

### 2.2. Inclusion Criterion

The main inclusion criterion was the level of disability < 5.0 measured with the EDSS. The score < 5.0 means that a patient is able to walk unassisted for at least 300 m [27].

### 2.3. Exclusion Criteria

Patients who reported non-MS-related gynecological, urological, or endocrine disorders that could affect sexual performance were excluded from the study. Patients with MS relapses or infectious diseases were also excluded. The absence of significant cognitive impairment was also verified with the medical records (MMSE > 27/30).

### 2.4. Ethics

Written informed consent was obtained from all patients. The study project was approved by the Bioethics Committee of the Medical University of Silesia.

### 2.5. Sexual Satisfaction Measurement

The SSQ was used for the assessment of SS [31]. The questionnaire consists of 10 items. Subjects provided their answers using a four-point scale (1: “I completely disagree,” 4: “I completely agree”). The consistency of the method measured by Cronbach's alpha is high (0.83). The authors of the method did not provide the cut-off point. The mean results for the healthy population range from 29.43 to 33.67 (min. 10, max. 40) [32, 33].

### 2.6. Other Clinical Variables

The MADRS was used to assess mood disorders. During a structured interview, patients responded to 10 items, which allowed the assessment of the severity of depressive symptoms in a 7-step scale for each item. The cut-off points include the following [34]: <9, symptoms absent; 9-17, mild depression; 18-34, moderate depression; and >34, severe depression.

The reliability of the scale measured with Cronbach's alpha in the Polish study is very high (0.90-0.94) [35].

The CISS questionnaire is used to diagnose coping styles in stressful situations and consists of 48 statements related to different behaviors typical of people in distress. Using a five-step scale, subjects assess the frequency with which they adopt a given behavior in stressful and difficult situations. The results are measured in three scales: task-oriented coping (TOC), emotion-oriented coping (EOC), and avoidance-oriented coping (AOC). The tool has a reliable version developed for the Polish population. Cronbach's alpha for the scales is 0.78-0.90 [29, 36].

MSISQ-19 is currently the most common tool used for the assessment of SD in PwMS [30, 37]. Using a five-point scale, subjects assess the frequency of the occurrence of particular problems. The tool includes three types of dysfunction, i.e., primary SD, which are the result of demyelinating lesions (sensory disturbances, paresthesia, erectile dysfunction, or insufficient vaginal lubrication), secondary SD not directly related to the genitals, which impair sexual intercourse (spasticity, pain, tremor, and sphincter dysfunction). Adverse drug reactions are also included in this type of dysfunction. Tertiary SD are the result of emotional, mental, social, and cultural factors that impair SS (feeling less attractive, less masculine or feminine, and fear of rejection).

Subjects assess the influence of symptoms on sexuality within the last six months (1: never, 2: rarely, 3: occasionally, 4: almost always, and 5: always). The scores of 4 and 5 are considered severe symptoms. Cronbach's alpha for the above scales is 0.82–0.87.

### 2.7. Statistical Analysis

The obtained results were analyzed using STATISTICA 10.0 PL (StatSoft Inc.) and the statistical software package R 3.1.2 (GNU GPL). The measurable data were characterized by the mean and standard deviation. The Shapiro-Wilk test was applied to verify the normality of distribution of variables with normal distribution. The significance of differences of the means was verified by the Student *t*-test for two groups or the ANOVA test for multiple groups. In the case of skew distributions, the normality of distribution in the groups was verified by *U* Mann–Whitney and Kruskal-Wallis tests. Percentage and the difference significance test for structure indicators were used for nominal data. The occurrence of the relationships between the nominal variable was checked with the chi-squared test (*χ*^2^ test) with corrections.

The models of multiple linear regression were used for the multivariable analysis. Variables significantly affecting the scores of SD, SS, and the relationship match were included as independent variables in multiple models.

The level of statistical significance of *p* < 0.05 and a low correlation with other predictors were adopted as the criteria for keeping the variable in the model. Multivariate linear regression model fitting was assessed by *R*^2^. The level of significance of <0.05 (SN: statistically nonsignificant) was used to verify the statistical hypothesis.

## 3. Results

No statistically important differences were found between men and women in the study group with respect to age, time from diagnosis, or the level of disability on the EDSS. The scores on MADRS were lower or equal to 17, which indicated no symptoms or mild symptoms of depression (Table 2).

The assessment of SD using MSISQ-19 indicated significantly higher values in primary and tertiary dysfunctions in the group of patients who were married or were in informal relationships (*p* < 0.01). In the same group, a higher prevalence of severe symptoms was reported (*p* < 0.05). Engaged patients reported SD the least frequently. We did not observe any influence of sex, the type of the disease, or previous treatment on the scores on the SD scale (Table 3).

The reported SD were significantly associated with the increase in disability on the EDSS, a higher score on MADRS, and the tendency to emotion-oriented coping style in difficult situations (*p* < 0.001). We also observed that the level of dysfunctions, especially primary and tertiary dysfunctions, significantly increased with age (*p* < 0.05) (Table 4).

The reported SS based on the SSQ questionnaire was on a relatively high level (mean 31 ± 6.2 points). No significant differences were found between males and females (Table 1). The highest SSQ results were observed in the case of patients living in cohabitation (*p* < 0.01).

The division into sex, the type of the disease, and immunomodulatory drugs did not differentiate SSQ values. On the other hand, high levels of SS were significantly associated with the low scores in MADRS (*p* < 0.01), lower CISS-EOS (emotion-oriented style) test results (*p* < 0.001), and higher CISS-TOS (task-oriented style) test results (*p* < 0.001). The prevalence of primary, secondary, and tertiary SD had a negative association with the level of reported SS (*p* < 0.001). However, we did not observe any correlation between disability on the EDSS and SS (Table 4).

Multifactorial linear regression models (Table 5) confirmed that SS was related to the lower scores on MADRS (*β* = −0.32, *p* < 0.05), higher CISS-TOC test results (*β* = 0.24, *p* < 0.001), and lower CISS-EOC test results (*β* = −0.19, *p* < 0.001). There was also a visible significant influence of disability level (EDSS) on the prevalence of secondary SD (*β* = 2.12, *p* < 0.001) and tertiary SD (*β* = 1.35, *p* < 0.001) and an increased number of severe symptoms (*β* = 1.09, *p* < 0.001). Emotion-oriented style CISS-EOC (*β* = 0.2, *p* < 0.001) proved to be relevant for all SD. Lowered mood significantly increased the number and intensity of tertiary SD (*β* = 0.38, *p* < 0.01), whereas age was associated with the number and the intensity of primary disorders (*β* = 0.18, *p* < 0.01).

## 4. Discussion

In the only meta-analysis that summarizes the relationship between MS and SD in women, the risk of SD increased by 1.87 among PwMS compared to the control group. Additionally, PwMS had lower levels by 2.41 of the mean total Female Sexual Function Index (FSFI) scores compared to the controls [6].

The studies on PwMS conducted in Turkey and Australia indicated that the occurrence of SD was more prevalent in women [17, 38]. The differences between males and females proved to be irrelevant in our analysis. The authors of the more recent studies presented similar conclusions [17, 39]. The American authors observed the difference only in tertiary dysfunctions [40].

The observed associations between the disability (measured on the EDSS) and the prevalence of primary, secondary (physical aspect), and tertiary SD (nonbiological aspect) were consistent with the majority of previous reports [9, 41, 42]. Different results were presented by Gruenwald et al. and Firdolas et al. Using the FSFI questionnaire for women, they did not present a clear correlation between the presence of sexual disorders defined by the FSFI and disability (EDSS) [43, 44]. Zorzon et al. explained these discrepancies most accurately in the follow-up study. After conducting the multiple regression analysis, the authors demonstrated that the correlation of SD with the result on the EDDS scale was no longer relevant after the elimination of psychological aspects [8]. Of note, the study by Demirkiran et al. emphasized that even 39% of patients without visible disability (EDSS < 2.0) reported SD [11]. The authors suggested that SD reported by patients with a low level of disability may occur due to the damage to the autonomic system, which is not assessed adequately by the scale.

It seems that the relation of SD and the quality of life of patients is best determined by SS, whose level measured by the SSQ questionnaire was not significantly lower in PwMS compared to healthy persons [31, 32, 38]. Additionally, we did not observe any significant differences between the level of SS in men and women. After comparing the SSQ results with the reported SD, a significant correlation was found for primary, secondary, and tertiary dysfunctions. Despite that, the dependence of SD and SS seems to be intuitive, and there are some reports that question the credibility of such dependence [45].

Of note, we found the lack of correlation of SS with the level of disability measured on the EDSS. Such dependence was not observed in men or women. DuPont was the first who noticed it and emphasized that SS in PwMS was higher than expected. However, he did not use any structured tool in the study. McCabe confirmed these observations and indicated that the level of SS in 381 patients was not significantly different from the healthy population [24, 46]. On the other hand, the findings of Hennesey et al. indicated that the presence of SD did not necessarily affect sexual activity. Even 61% of women who reported SD were satisfied with the level of their sexual activity [47].

Currently, it is stressed that MS does not have to be a risk factor for SD in itself, but the coexisting psychological, emotional, social, or cultural problems could lead to SD [6]. It seems appropriate to search for the factors that have the influence on SS in MS patients irrespective of the objectively measured disability (EDSS) or reported SD. Other factors, such as lowered mood, cognitive functioning, stress coping strategies, and the quality of the partner relationship, have been discussed in the literature [8, 24].

The issue of the relation between lowered mood on MADRS and the prevalence of SD has been discussed many times in the literature [8, 11, 41, 48, 49]. People with MS who are depressed might not search for sexual intimacy, and conversely, patients with MS-related SD might experience reactive depression [12]. Our study demonstrated that in most patients, the MADRS score did not show any significant depressive disorders. However, the higher the score on MADRS, the lower the reported SS was.

Another factor we assessed was related to stress coping strategies. In MS patients, stress is not only associated with breakthrough moments, such as diagnosis or relapse, but it is present in everyday situations when they have to cope with disability.

In a few studies assessing the relationships between stress coping strategies in MS patients and SS, the authors showed that the applied strategies were significant predictors of the quality of life of MS patients [50, 51]. In the study involving over 100 MS patients, Mohr et al. observed that together with an increase in disability, an increase in the use of maladaptive emotional-oriented strategies and avoidance-oriented strategies was noted. This also correlated with higher scores on depression scales [52]. McCabe and McKern emphasized that especially wishful thinking, which is a type of emotional-oriented style, strongly correlated with the lowering of the quality of life and decreased SS [24, 50]. Our study confirmed the decreased SS in patients with emotional-oriented strategy. The highest level of SS was reported by patients with the task-oriented style. It would be useful to bear in mind these problems and discuss them, as it is proved that proper psychoeducation results in the improvement of SS for both partners [53]. Many patients are not aware of the fact that their problems associated with intimacy can be treated. Zivadinov et al. believe that insufficient attention paid to the sphere of sexual activity can result from a small number of the spinal cord MRI [42]. Detecting plaques in this part of the nervous system could prompt a neurologist to discuss possible sexual problems with a patient.

## 5. Conclusions

The level of SS in MS patients with the EDSS score below 5.0 points was not significantly lower.

SS depends on reported SD and lowered mood, and it was not significantly related to the level of disability in patients with the EDSS score < 5.0. Patients with the task-oriented coping style reported the highest level of SS, while patients with emotion-oriented style reported the lowest level of SS. We did not observe any significant differences in SS between males and females.

## 6. Limitations

Care should be exercised when formulating the conclusion on the cause-and-effect relationships due to a retrospective character of the study. We did not correlate SD in MS patients with the occurrence of plaques in the brain or the spine on MRI. We did not assess fatigue or autonomic dysfunction in MS patients. We did not correlate them with SD and SS. We did not compare the effect of different immunomodulatory drugs on the sexual function.

The choice of a tool for the assessment of SS was challenging. There are some tools used for assessing SS in the English literature. The FSFI was used in several studies. However, it is only applicable to women. The Index of Sexual Satisfaction (ISS), the Global Measure of Sexual Satisfaction (GMSEX), and New Sexual Satisfaction Scale-Short (NSSS-S) can be applied to both sexes. However, the authors could not access Polish language versions of these questionnaires. SSQ applied by the authors was validated on a relatively small group. High expectations can be associated with the Questionnaire of Sexual Satisfaction by Plopa, as it was validated on a group of 3,000 people. However, this tool was not accessible when the study was planned [54].

We also know that lesbian, gay, bisexual, and transgender (LGBT) patients might experience unique difficulties with MS related to their sexual orientation [55]. But our group was too small to make reliable comparisons. With the confidence level of *α* = 0.95, the margin of error was 11%.

## Figures and Tables

**Table 1:** Level of sexual satisfaction (SSQ) in the study group including personal and environmental data, the type of the disease, and treatment.

SSQ [10 ÷ 40]		Total *N* = 70 (100%)	
		31 ± 6.2	
		*X* ± *S*	*p* value
Sex	Females	30.8 ± 6	NS (*p* = 0.5)
	Males	31.3 ± 6.7	

Partner relationship	Marriage^1^	30.8 ± 5.8	**p** < 0.01
	Engagement^2^	34.7 ± 0.6	
	Cohabitation^3^	35.9 ± 5.1∗^**4**^	
	Free relationship^4^	26.8 ± 7.9∗^**3**^	

Type of the disease	Relapsing-remitting MS	30.9 ± 6.2	NS (*p* = 0.09)
	Progressive-relapsing MS	38 ± 0	
	Primary progressive MS	27.5 ± 6.6	
	Secondary progressive MS	29.7 ± 6	

Previous treatment	Immunomodulation (interferons, glatiramer acetate, and fingolimod)	32.1 ± 5.7	NS (*p* = 0.86)
	Symptomatic treatment	31.3 ± 6.7	

*X* ± *S*: mean and standard deviation. ∗*p* < 0.05.

**(a):** 

	Total *n* (%)		Females *n* (%)	Males *n* (%)	*p* value
	76 (100)		46 (60.5)	30 (39.5)	
	min ÷ max	*X* ± *S*	*X* ± *S*	*X* ± *S*	
Age (in years)	21 ÷ 54	35.2 ± 7.8	35.1 ± 6.5	35.2 ± 9.5	SN (*p* = 0.86)
Time from diagnosis (in years)	1 ÷ 31	6.8 ± 4.6	6.6 ± 3.2	7.0 ± 6.3	SN (*p* = 0.40)
Expanded Disability Status Scale (EDSS) [1 ÷ 10]	1 ÷ 4.5	3.2 ± 1.4	3.0 ± 1.1	3.1 ± 1.5	SN (*p* = 0.40)
Montgomery-Asberg Depression Scale (MADRS) [0 ÷ 60]	2 ÷ 17	9.8 ± 4.3	9.6 ± 4.0	10.2 ± 4.8	SN (*p* = 0.69)

**(b):** 

		Total *n* (%)	Females *n* (%)	Males *n* (%)	*p* value
Type of the disease	Relapsing-remitting MS	65 (85.5)	41 (89.1)	24 (80.0)	SN (*p* = 0.61)
	Progressive-relapsing MS	3 (3.9)	1 (2.2)	2 (6.7)	
	Primary progressive MS	5 (6.6)	3 (6.5)	2 (6.7)	
	Secondary progressive MS	3 (3.9)	1 (2.2)	2 (6.7)	

Previous treatment	Immunomodulation (interferons, glatiramer acetate, and fingolimod)	65 (85.5)	41 (89.1)	24 (80.0)	SN (*p* = 0.86)
	Symptomatic treatment	11 (14.5)	5 (10.9)	6 (20.0)	SN (*p* = 0.68)

Partner relationship	Marriage	47 (61.8)	30 (65.2)	17 (56.7)	SN (*p* = 0.30)
	Engagement	3 (3.9)	0 (0.0)	3 (10.0)	
	Cohabitation	11 (14.5)	7 (15.2)	4 (13.3)	
	Free relationship	9 (11.8)	6 (13.0)	3 (10.0)	
	Not involved in any relationship	6 (7.9)	3 (6.5)	3 (10.0)	

*X* ± *S*: mean and standard deviation.

**Table 3:** Reported sexual dysfunctions (MSISQ-19) including personal and environmental data, the type of the disease, and treatment.

MSISQ-19 Total *N* = 70 (100%)		Primary [5 ÷ 25]		Secondary [9 ÷ 40]		Tertiary [5 ÷ 25]		Severe symptoms [0 ÷ 19]	
		12.6 ± 5.4		22.1 ± 7.2		12.8 ± 5.8		4.7 ± 4.6	
		*X* ± *S*	*p* value	*X* ± *S*	*p* value	*X* ± *S*	*p* value	*X* ± *S*	*p* value
Sex	Females	13.3 ± 5.4	NS (*p* = 0.17)	21.7 ± 6.4	NS (*p* = 0.75)	12.6 ± 5.5	NS (*p* = 0.71)	4.3 ± 4.4	SN (*p* = 0.49)
	Males	11.5 ± 5.2		22.7 ± 8.3		13.3 ± 6.3		5.2 ± 4.9	

Partner relationship	Marriage^1^	13.6 ± 5.2^∗^^**2**^	**p** < 0.01	23 ± 6.4	NS (*p* = 0.06)	13.2 ± 5.4^∗^^**2**^	**p** < 0.05	5 ± 4.4^∗^^**2**^	**p** < 0.05
	Engagement^2^	5 ± 0^∗^^**1,4**^		17.3 ± 1.2		7.7 ± 2.3^∗^^**1,4**^		1.3 ± 0.6^∗^^**1,4**^	
	Cohabitation^3^	8.8 ± 4.1		17.3 ± 7.2		8.9 ± 4.3		1.5 ± 2.3	
	Free relationship^4^	14.4 ± 4.3^∗^^**2**^		25.6 ± 9.4		17.2 ± 6.4^∗^^**2**^		7.9 ± 5.3^∗^^**2**^	

Type of the disease	Relapsing-remitting MS	12.3 ± 5.5	NS (*p* = 0.61)	21.7 ± 7.2	NS (*p* = 0.76)	12.5 ± 5.7	NS (*p* = 0.46)	4.4 ± 4.3	SN (*p* = 0.58)
	Progressive-relapsing MS	14.7 ± 0.6		25 ± 1.7		16 ± 5.2		7.7 ± 4.9	
	Primary progressive MS	14.8 ± 5.1		23 ± 5.8		13.8 ± 5.9		6 ± 5.2	
	Secondary progressive MS	13.7 ± 7.2		26 ± 12.3		15.7 ± 8.1		6.3 ± 8.5	

Previous treatment	Immunomodulation (interferons, glatiramer acetate, and fingolimod)	13.5 ± 5.2	NS (*p* = 0.3)	24.6 ± 7.7	NS (*p* = 0.18)	14.5 ± 4.9	NS (*p* = 0.13)	6.1 ± 4.6	SN (*p* = 0.16)
	Symptomatic treatment	14.5 ± 4.6		24.4 ± 6.8		14.9 ± 5.8		6.5 ± 5.6	

*X* ± *S*: mean and standard deviation. ∗*p* < 0.05.

**Table 4:** Correlation of sexual dysfunction, sexual satisfaction, and relationship quality with age and time from diagnosis and psychophysical status (Spearman's rank correlation coefficient).

Scales (pts)	Age (in years)	Time from diagnosis (in years)		Psychophysical assessment				
			SSQ [10 ÷ 40]	EDSS [1 ÷ 10]	MADRS [0 ÷ 60]	CISS-TOC [16 ÷ 80]	CISS-EOC [16 ÷ 80]	CISS-AOC [16 ÷ 80]
Primary [5 ÷ 25]	0.25^∗^	0.06	-0.54^∗∗∗^	0.25^∗^	0.29^∗∗^	-0.09	0.56^∗∗∗^	0.18
Secondary [9 ÷ 40]	0.17	0.12	-0.37^∗∗∗^	0.40^∗∗∗^	0.27^∗^	-0.05	0.45^∗∗∗^	0.23^∗^
Tertiary [5 ÷ 25]	0.27^∗^	0.18	-0.47^∗∗∗^	0.50^∗∗∗^	0.47^∗∗∗^	0.08	0.49^∗∗∗^	0.22
Severe symptoms [0 ÷ 19]	0.19	0.07	-0.41^∗∗∗^	0.37^∗∗∗^	0.31^∗∗∗^	-0.04	0.45^∗∗∗^	0.16
SSQ [10 ÷ 40]	-0.15	0.03	1.00	-0.10	-0.35^∗∗^	0.30^∗∗∗^	-0.44^∗∗∗^	-0.16

^∗^ *p* < 0.05, ^∗∗^*p* < 0.01, and ^∗∗∗^*p* < 0.001.

**Table 5:** Model of multiple regression for factors affecting sexual satisfaction.

Parameter	Independent variable	*β*	*β* 95% CI	*R* ^2^	*p* value
Primary [5 ÷ 25]	Age (in years)	0.18^∗∗^	0.12 ÷ 0.25	0.38	*p* < 0.0001
	CISS-EOC [16 ÷ 80]	0.24^∗∗∗^	0.2 ÷ 0.28		

Secondary [9 ÷ 40]	EDSS [1 ÷ 10]	2.12^∗∗∗^	1.63 ÷ 2.62	0.34	*p* < 0.0001
	CISS-EOC [16 ÷ 80]	0.24^∗∗∗^	0.19 ÷ 0.29		

Tertiary [5 ÷ 25]	EDSS [1 ÷ 10]	1.35^∗∗∗^	0.96 ÷ 1.74	0.47	*p* < 0.0001
	MADRS [0 ÷ 60]	0.38^∗∗^	0.26 ÷ 0.5		
	CISS-EOC [16 ÷ 80]	0.2^∗∗∗^	0.16 ÷ 0.23		

Severe symptoms [0 ÷ 19]	EDSS [1 ÷ 10]	1.09^∗∗^	0.76 ÷ 1.41	0.31	*p* < 0.0001
	CISS-EOC [16 ÷ 80]	0.16^∗∗∗^	0.13 ÷ 0.19		

SSQ [10 ÷ 40]	Partner relationship Marriage/engagement/cohabitation/free relationship	-0.93^∗^	−1.38 ÷ −0.48	0.31	*p* < 0.0001
	MADRS [0 ÷ 60]	-0.32^∗^	−0.47 ÷ −0.18		
	CISS-TOC [16 ÷ 80]	0.24^∗∗∗^	0.17 ÷ 0.3		
	CISS-EOC [16 ÷ 80]	-0.19^∗∗∗^	−0.24 ÷ −0.14		

^∗^ *p* < 0.05, ^∗∗^*p* < 0.01, and ^∗∗∗^*p* < 0.001.

## Data Availability

Data will be accessible on author ResearchGate profile.
